# Supplementary material for: Scales of transformations—Modelling settlement and land-use dynamics in late antique and early medieval Basel, Switzerland
Source: PLoS One. 2023 Feb 1;18(2):e0280321. doi: 10.1371/journal.pone.0280321 (PMC9891536; doi:10.1371/journal.pone.0280321)
Supplement: Attachment — Submitted filename: cover_response_R1.odt [file pone.0280321.s001.odt]

Dear Reviewers,

Many thanks for the productive suggestions regarding our article! In the following, we want to emphasize the few changes to the original draft and answer the issues raised by the two reviewers.
We changed the title according to suggestions by Reviewer 2. We now refer to the modelling character of the paper and the settlement and land-use spatial analysis. 
We included a short description of the variables of the model (e.g. Xelev) in section 2.4.2. We hope that it becomes more clear now. We already solved all the minor issues raised by Reviewer 2. 
Overall, we are extremely grateful for the very positive feedback on the paper and particularly on the model. We are looking forward to further explore the run-off model and integrate a continental-wide approach in a future project! We included both reviewers into the acknowledgement section of the paper!!

Point-by-point response to reviewers:
Rev#1:
Many thanks for this very positive review! We are delighted to see how the model, the workflow, and the results were appreciated! 
Rev#2:
Again, many thanks for this equally positive and constructive review! The minor changes raised here will be listed and answered in the following:
Title change: as described in the general comments above, we changed the title to better fit the paper. You were right that a too general title does not actually refer to the modelling approach and the case study!
Variable description: we described the variables used in the model, like elevation, slope and others. We initially thought that they were self-describing but you were completely right that in formulas, this is not always the case! We hope that now it becomes cleared what are the input variables. Many thanks for this!
Minor issues:
Sentence corrected
Footnote included with description
Sentence corrected
Known corrected
Former line 218: n(late antiquity) refers to the occupation time of Aeschenvorstadt, which runs over a long period, including also Early Medieval period burials. Here, we emphasize the late antique occupation. 
Museum of Basel, corrected in the MS
Space put
Wording checked and corrected (typo)
Wording corrected and replaced harmonized
Sentence revised
Wording revised


Many thanks again for taking time to review the manuscript!
Best regards,

Michael and Margaux
Kiel/Basel, the 21st of October 2022


For the revision R1, we used the template and hence there is no track-changes version of the MS. We hope, however, that all minor changes are visible in the MS (e.g. the section model set-up (formerly 2.4.2). 
The PLOS ONE system further requested copyrights for the images 1, 2, 3, however the images do not contain particularly protected spatial information and are only reused visualizations. 
Eventually, we confirm the updated funding statements:
MK received funding from the European Commission and the Masaryk University, Brno; Czech Republic, grant number CZ.02.2.69/0.0/0./18_053/0016952; Postdoc2MUNIm order number 21 0053
MK received funding from the German Research Foundation (DFG) and the CRC1266 Scales of Transformation, Kiel University, grant number 290391021
MLCD received funding from the University of Basel and the Swiss National Science Foundation (SNSF).
Many thanks again for considering our work for publication in PLOS ONE and we are looking forward to the publication process!

With best regards

Michael Kempf and Margaux Depaermentier,

Nice/Basel, the 14th of October 2022
